# Rethinking catalysis: interpretable AI and description of real-world conditions *via* materials genes

**DOI:** 10.1039/d5fd00137d

**Published:** 2026-02-06

**Authors:** Lucas Foppa, Matthias Scheffler

**Affiliations:** a The NOMAD Laboratory at the Fritz Haber Institute of the Max Planck Society Berlin Germany foppa@fhi-berlin.mpg.de; b Molecular Simulations from First Principles e.V. Berlin Germany

## Abstract

Descriptors link basic physicochemical parameters that characterize the materials and the environment to the catalytic performance. Traditionally, descriptors are rooted in mechanistic understanding of elementary surface reactions gained from surface science and atomistic simulations on well-defined surfaces and under vacuum. However, real-world catalysis operates under elevated pressures and temperatures, where an intricate interplay of multiple physical processes, including significant materials' restructuring and transport phenomena, governs performance. To bridge this gap, we introduced an interpretable artificial intelligence (AI) approach that identifies key physicochemical parameters correlated with the measured catalytic performance. Analogous to genes in biology and medicine, these “materials genes” provide a statistical description of catalysis without requiring the explicit atomistic description of the underlying physical processes. Here, we combine the sure-independence-screening-and-sparsifying-operator (SISSO) symbolic-regression AI approach with a sensitivity analysis based on partial derivatives to determine the most influential genes needed to describe the selectivity of supported palladium-based metal alloy nanoparticles in the hydrogenation of concentrated acetylene streams. The identified genes include the calculated average d-band center and the measured average particle diameter, indicating the crucial role of adsorption and structure sensitivity on the formation of ethylene.

## Abstract 2

We introduce an interpretable artificial intelligence approach that identifies key physicochemical parameters correlated with the measured catalytic performance.

## Graphical abstract

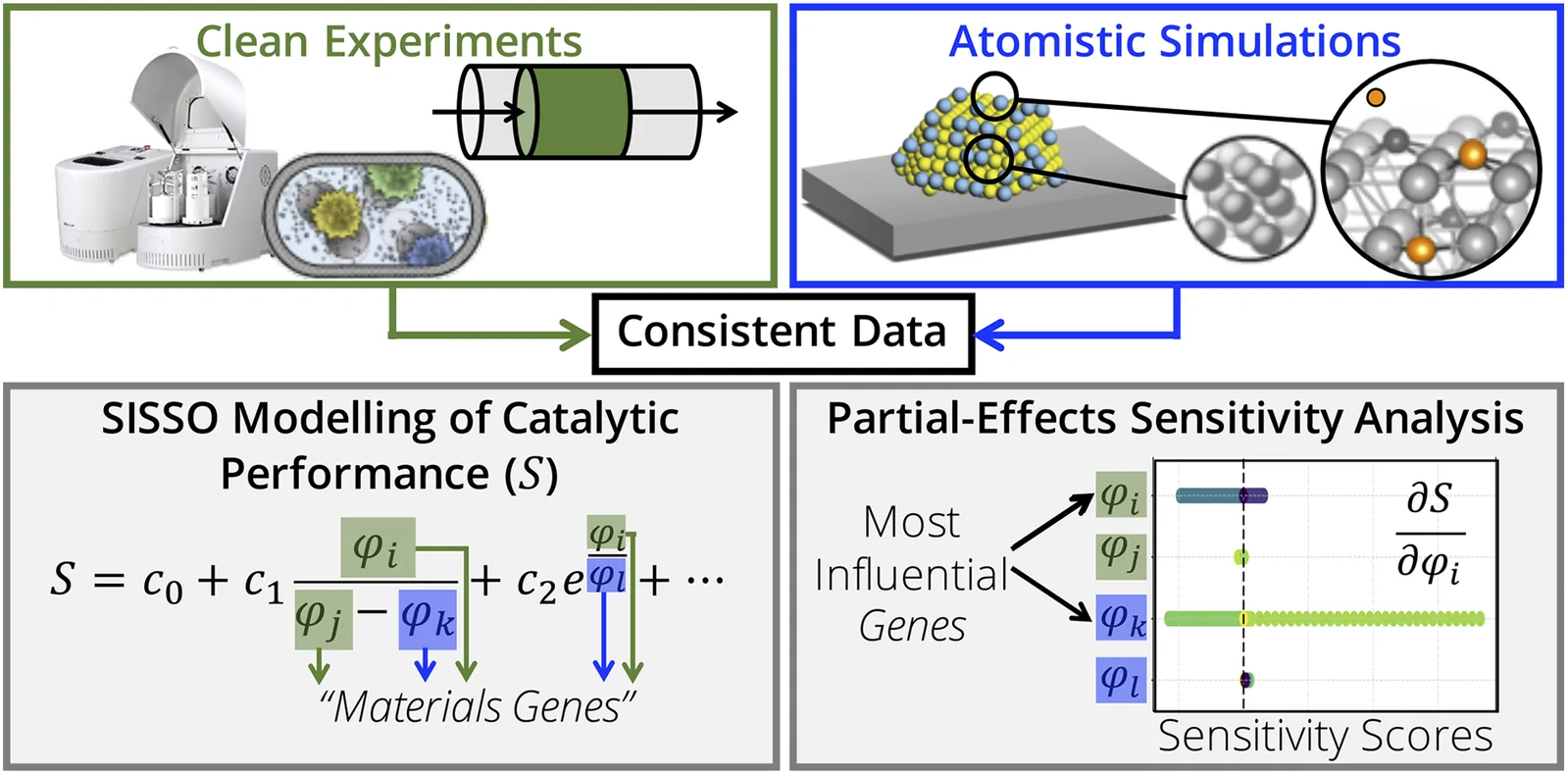

## 1 Introduction

**Fig. 1:**
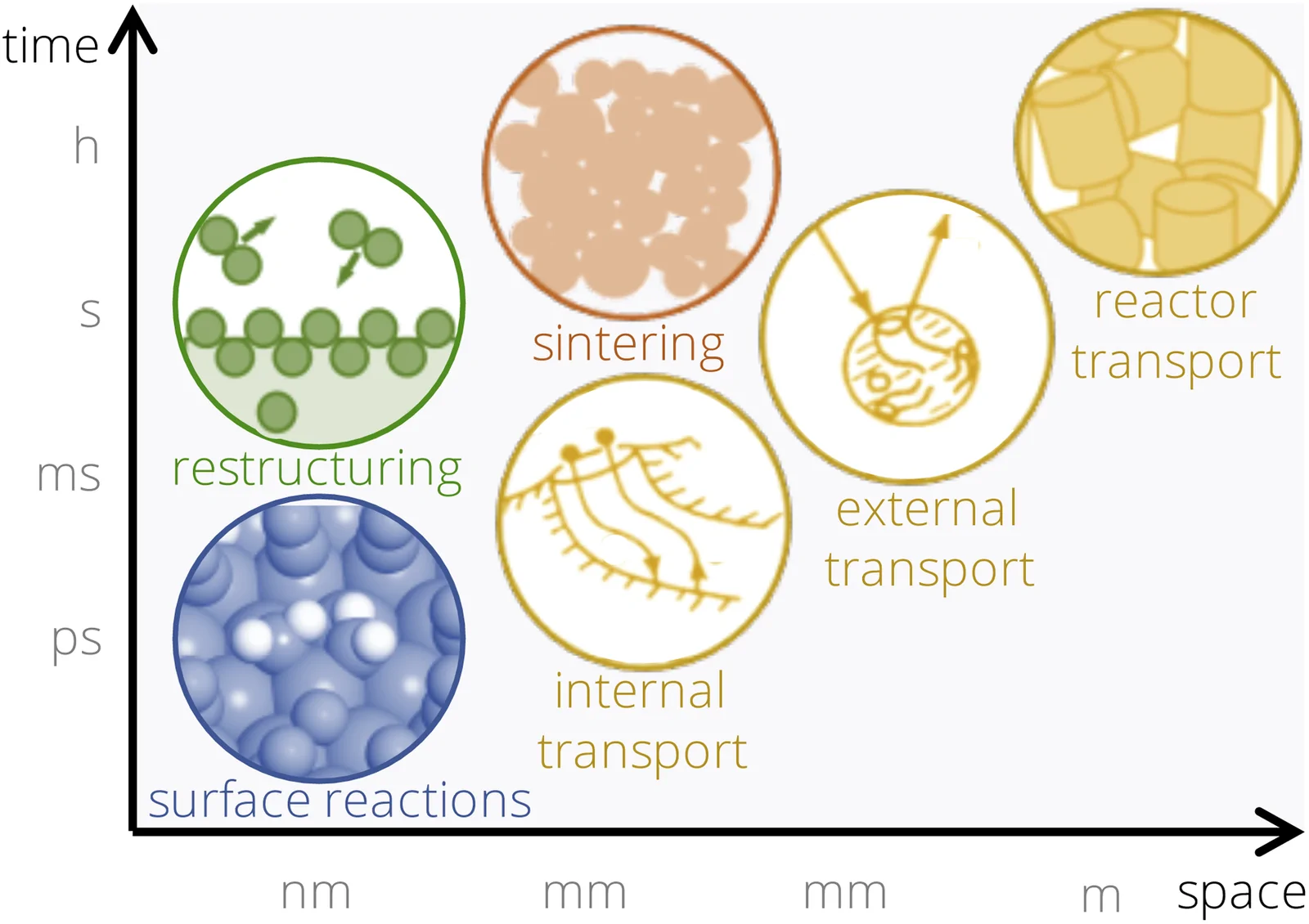
The performance of real-world heterogeneous catalysts is governed by an intricate interplay of multiple physical processes occurring at extremely different time and length scales. The solid-state chemistry of the material and its restructuring, for instance, is coupled with chemistry of the catalytic reaction, which depends on the surface reaction networks.

Progress in catalyst design depends on identifying basic physicochemical parameters that characterize materials and correlate with their catalytic performance,^1,2^ a task complicated by the extensive range of variables that can be tuned for creating a new material. These parameters, or functions thereof, establish quantitative relationships with catalytic-performance metrics such as activity or selectivity. We refer to the functions of these physicochemical parameters as descriptors hereafter. Traditionally, descriptors are rooted in fundamental understanding of the physical processes governing catalysis. Surface science^3–8^ and *ab initio* atomistic simulations^9–11^ provide detailed information on adsorption and elementary reactions occurring on well-defined model surfaces under vacuum and low temperatures. This knowledge led to descriptors based on adsorption energies of surface intermediates involved in important steps of the catalytic cycle.^12^ Adsorption energies offer an intuitive picture of heterogeneous catalysis, as they embody the Sabatier principle^13^ of optimal binding strength. They also relate to activation energy barriers through Brønsted–Evans–Polanyi relationships^14,15^ and to basic electronic properties of the material through models such as the d-band theory.^16–18^ The use of adsorption-energy-based descriptors is convenient for screening new materials,^19^ since such quantities can be computed using density functional theory (DFT) calculations. Machine-learning interatomic potentials trained on DFT data further accelerated the screening of materials based on adsorption-energy descriptors.^20^ However, descriptors derived from idealized model systems such as adsorption energies are often insufficient to describe the catalytic performance observed under realistic, high-performance operating conditions, characterized by high pressures and temperatures.^21^ This is because under such realistic conditions, the catalytic performance is governed by an intricate interplay of many physical processes (Fig. 1).^7,8,22,23^ These include not only large and interconnected networks of surface elementary reactions, but also the catalyst's dynamic restructuring and changes in the surface composition during the reaction, mass and heat transport, particle sintering, to name a few. Notably, these physical processes operate across very different time and length scales and are often entangled. Therefore, explicitly modeling the full catalytic progression in order to design a new material is deemed inappropriate. Instead, our goal is to describe the statistical correlation of key physicochemical parameters with the experimental catalyst performance.

Artificial intelligence (AI) offers the strategy for identifying correlations between physicochemical parameters and the measured catalytic performance.^12,24–28^ However, the utilization of experimental data in AI frameworks is complicated by the lack of consistent and well-characterized datasets, *i.e.*, datasets created according to rigorous and standardized procedures.^29–32^ To address these limitations, we have developed an interpretable AI approach designed to identify physically meaningful descriptors using clean experimental data.^2,29^ The term “clean data” refers to the fact that the materials considered in the AI analysis are carefully synthesized, characterized, and tested according to standardized and well annotated experimental procedures. Indeed, the properties of the material and its catalytic performance are often sensitive to details of the experimental workflow, such as the sequence of synthesis steps and the pretreatment utilized prior to performance testing. Thus, all relevant steps of the workflow should be recorded in detail in order to guarantee the reproducibility of the experiment as well as the consistency of the resulting data.^29–32^ Unlike traditional physics-based models and atomistic simulations, this approach focuses on discovering statistical correlations rather than explicitly simulating all the physical processes, thereby leaving it open what structure or composition the working catalyst has and capturing the complexity of catalysis more effectively. Within this approach, a large set of experimentally measured or calculated candidate descriptive parameters, termed primary features, is first chosen. The primary features characterize the materials as well as the reaction environment, *e.g.*, the temperature, pressure, or chemical potential of the phase in which reactants and products are contained. They correlate with physical processes that might be relevant for the system under consideration. Then, AI is utilized to identify a smaller number of *key* primary features correlated with the measured catalytic performance, from all initially offered primary features. Crucially, we note that AI identifies potentially nonlinear relationships among multiple primary features. In analogy with biology and medicine, these key primary features are termed materials genes of the catalytic function of interest, *e.g.*, the activity or selectivity.^2^ Thus, the descriptor is a (nonlinear) function of the materials genes. The materials genes correlate with crucial physical processes that trigger, facilitate, or hinder the catalytic function of interest. In view of the high intricacy of these processes, the microscopic relationship between these genes and the catalytic function of interest might remain unknown. This concept has been applied to model the catalytic performance in alkane oxidation,^2,33,34^ CO oxidation,^35^ CO_2_ hydrogenation^36^ and selective hydrogenation of concentrated acetylene streams.^37^

The Sure Independence Screening and Sparsifying Operator (SISSO) symbolic-regression method^38,39^ gained prominence as a systematic AI approach to identify descriptors in materials science and catalysis.^40–44^ SISSO is well suited for the identification of materials genes in consistent experimental datasets, which often contain a rather small number of materials, *e.g.*, <10^2^, compared to the number of data points typically used to train AI and machine-learning models, *e.g.*, >10^4^. The descriptors identified by SISSO are interpretable in the sense that they depend on a (small) subset of physically meaningful genes, selected from many offered primary features. Additionally, the mathematical relationship between the genes and the target is explicit. Indeed, symbolic-regression methods identify analytical expressions describing the correlations in data.^45–47^ Such interpretability facilitates the extraction of physical insights beyond mere prediction.

**Fig. 2:**
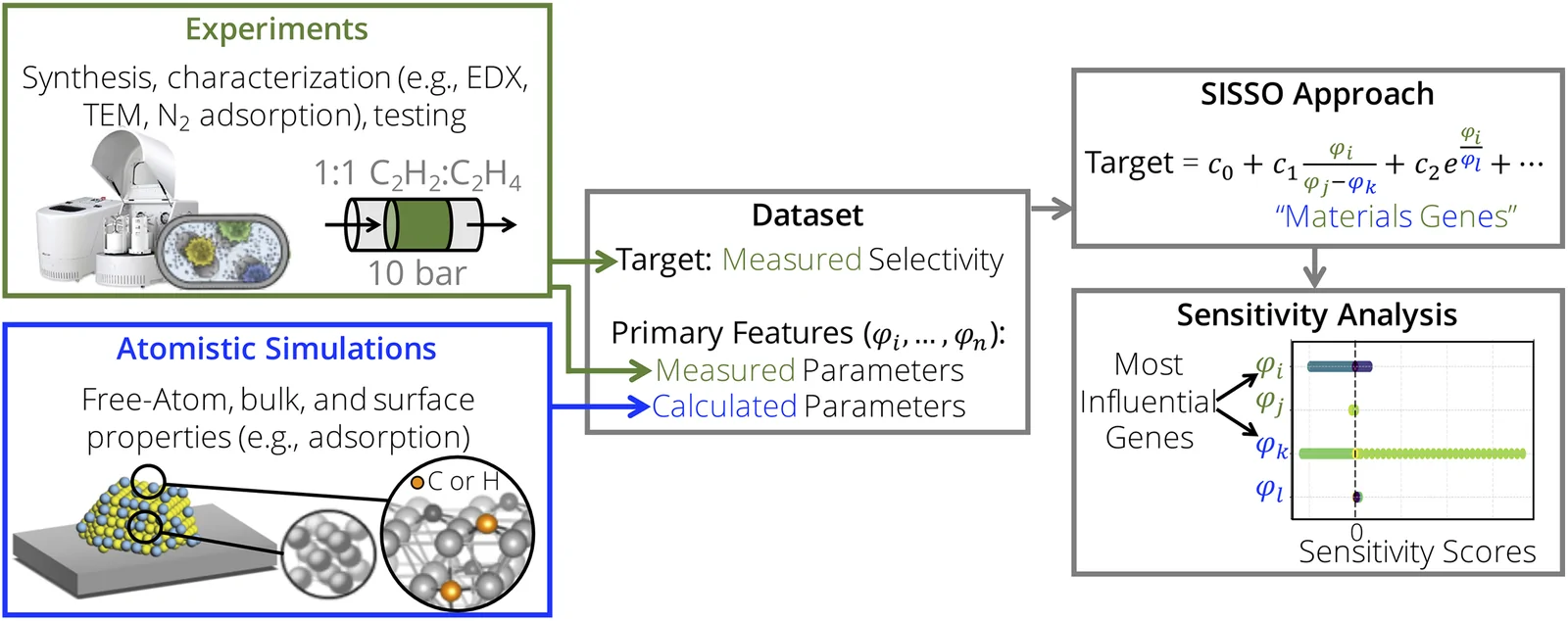
Interpretable AI approach for the identification of descriptors for heterogeneous catalysis based on data measured experimentally and calculated by atomistic simulations. By using consistent experiments and atomistic simulations on model systems, we create a dataset containing a target material’s function of interest (*e.g.*, selectivity) and many *candidate* descriptive parameters (also called primary features) that characterize the materials and possibly relevant underlying physical processes. Then the sure-independence screening and sparsifying operator (SISSO) symbolic-regression approach identifies analytical expressions correlated with the target and depending on only a few of the initially offered primary features. These selected primary features are termed materials genes. Finally, the partial effects (PEs) sensitivity analysis pinpoints the genes that most influence the SISSO model. Parts of the figure are adapted from ref. 37.

In this work, we complement the SISSO approach with a gradient-based partial-effects (PEs)^48,49^ sensitivity analysis to identify descriptors for heterogeneous catalysis and quantify the influence of genes selected in the descriptor expressions (Fig. 2). The approach is illustrated for supported metal nanoparticles (NPs) applied in the selective hydrogenation of concentrated acetylene streams.^37^ The experimental data utilized here were created and analyzed by a focused SISSO approach in a previous publication.^37^ Here, we utilize a SISSO model for time-dependent selectivity towards ethylene identified in this previous study in order to demonstrate how the PE sensitivity analysis identifies the most influential genes of a SISSO model thus providing detailed physical insight per material.

## 2 Methods

### 2.1 Dataset

The dataset analyzed in this publication can be found in ref. 37. We analyze metal NPs supported on high-surface-area-α-Al_2_O_3_. These catalysts were synthesized *via* mechanochemistry,^50–53^ which is a promising, more atom-efficient and environmental friendly alternative to wet synthesis methods.^54^ Nine palladium-based bimetallic alloy NPs are considered: Pd_1_Ag_1_, Pd_1_Ag_5_, Pd_1_Ag_9_, Pd_1_Au_1_, Pd_1_Au_5_, Pd_1_Au_9_, Pd_1_Cu_1_, Pd_1_Cu_5_, Pd_1_Cu_9_. These materials were characterized prior to the reaction by energy dispersive X-ray analysis in a scanning electron microscope (EDX-SEM), transmission-electron microscopy (TEM), and N_2_ physisorption. They were tested in the selective hydrogenation of highly concentrated acetylene streams. The reaction was performed in a steel-lined continuous-flow fixed-bed reactor at 10 bar and 150 °C. The applied feed contained a C_2_H_2_ : C_2_H_4_ : H_2_ ratio of 1 : 1 : 5 at a weight hourly space velocity of 90 000 cm^3^ g_cat_^−1^ h^−1^. The feed contained equimolar acetylene–ethylene mixtures, which would result from a hypothetical electric plasma-assisted methane-to-ethylene process. This plasma process can enable the production of ethylene from natural gas, biogas, or hydrogenated CO_2_ using the short-term surpluses in electricity from renewable sources.^55–57^ In this process, acetylene is formed as a by-product in equimolar concentrations and it needs to be selectively converted to ethylene in a dedicated, separate downstream process.^58^ Note that the selective hydrogenation of concentrated acetylene streams (>14 vol%) is a significantly different catalytic process compared to the selective hydrogenation of acetylene traces (between 0.1 and 2 vol%), which has been investigated much more extensively.^55,59–61^ Detailed descriptions of the procedures for materials synthesis, characterization, and testing are available elsewhere.^37^

As an example of target catalytic performance, we analyze the selectivity towards ethylene, denoted *S*_C_2_H_4__. This selectivity was monitored during time on stream (*t*_OS_). The dataset contains 539 measurements of *S*_C_2_H_4__, corresponding to 9 materials measured at multiple *t*_OS_'s between 0 and 400 minutes (min). We note that the measured *S*_C_2_H_4__ changes significantly with *t*_OS_ for some of the materials (see below). The SISSO modelling aims at describing *S*_C_2_H_4__ for different materials as well as its evolution with *t*_OS_ during the initial stages of the catalytic process. All *S*_C_2_H_4__ values correspond to materials and reaction conditions resulting in full (100%) conversion of the reactant acetylene. Thus, the selectivity values can be compared, as they correspond to a fixed conversion of acetylene.

As primary features, we utilized a combination of experimentally measured parameters with parameters calculated using atomistic simulations (Table 1). Four primary features were obtained from the experimental characterization of the materials:^53,62^ the metal loading (*w*_metal_), the mean and standard deviation of the particle-diameter distribution (*D*_*µ*_ and *D*_*σ*_), and the specific surface area (*s*_BET_). These primary features correspond to the entire material (NP + support) and they relate metal–support interactions and mesoscale properties of the materials. Noteworthy, these materials' properties are measured before the material is exposed to the reaction environment. Under the reaction, it is possible or even likely that properties, *e.g.*, those related to particle-size distribution, change. In addition to the primary features extracted from the characterization of the materials, we included 3 experimental elemental free-atom properties and 3 experimental bulk properties as primary features reflecting the chemistry of the NP bulk, such as the ionization potential (IP) and the closest interatomic distance (*d*_closest_). Those primary features were obtained from tabulated data.^63–65^*t*_OS_ is also offered as a primary feature in order to capture the time-on-stream dependent behavior of *S*_C_2_H_4__.

**Table 1:** Primary features offered in the SISSO analysis. These parameters were measured experimentally or calculated with DFT-GGA. They characterize the materials and reaction conditions and they may correlate with possible underlying physical processes

Type	Name	Symbol	Unit	Method
Reaction condition	Time on stream	*t*_OS_	min	—

NP + support	Total metal loading (weight fraction)^b^	*w*_metal_	—	EDX-SEM^53,62^
NP + support	Mean value of particle-diameter distribution^b^	*D*_*µ*_	Å	TEM^53,62^
NP + support	Standard deviation of particle-diameter distribution^b^	*D*_*σ*_	Å	TEM^53,62^
NP + support	Specific surface area^b^	*s*_BET_	m^2^ g^−1^	N_2_ adsorption^53,62^

NP free-atom	Ionization potential^a^	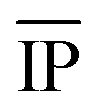	eV	Experimental^63^
NP free-atom	Electron affinity^a^	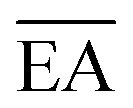	eV	Experimental^64^
NP free-atom	Pauling electronegativity^a^	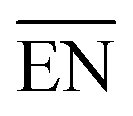	—	Experimental^64^

NP bulk^a^	Closest interatomic distance^a^	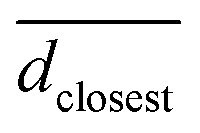	Å	Experimental^65^
NP bulk^a^	Cohesive energy^a^	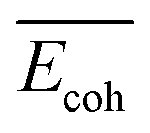	eV per atom	Experimental^65^
NP bulk^a^	Bulk modulus^a^	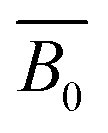	GPa	Experimental^65^

NP clean surface^b^	Energy of the d-band center^a^	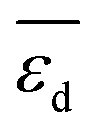	eV	DFT-GGA^66^

NP surface with C^b^	Critical surface carbon chemical potential^a^	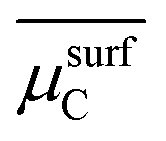	eV	DFT-GGA^67^
NP surface with C^b^	Critical subsurface carbon chemical potential^a^	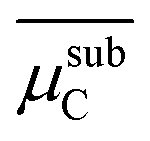	eV	DFT-GGA^67^
NP surface with C^b^	Distance expansion due to subsurface carbon^a^	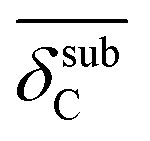	Å	DFT-GGA^67^
NP surface with C^b^	Surface deformation energy due to subsurface carbon^a^	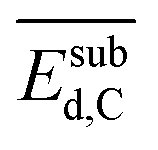	eV	DFT-GGA^67^
NP surface with C^b^	Subsurface carbon binding energy^a^	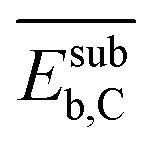	eV	DFT-GGA^67^
NP surface with H^b^	Surface binding energy^a^	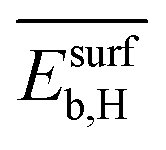	eV	DFT-GGA^66^
NP surface with H^b^	Subsurface binding energy^a^	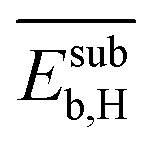	eV	DFT-GGA^66^
NP surface with H^b^	Work-function change due to hydrogen adsorption^a^	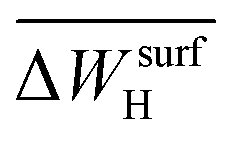	eV	DFT-GGA^66^

a Composition averages.

b Measured prior to applying the material in the reaction.

The 9 primary features calculated with atomistic simulations reflect the properties of the pristine NP surfaces and the interaction of carbon and hydrogen with the surface and subsurface. These primary features were calculated by DFT with the generalized gradient approximation for electron exchange and correlation (DFT-GGA) considering well-defined low-index model surfaces of previous works.^66,67^ The face-centered-cubic crystal structure and its (111) surface was adopted for palladium, silver, and gold. The properties associated with the most stable surface adsorption sites, *i.e.*, the adsorption site corresponding to the highest binding strength, were considered. Examples of calculated primary features are the energy of the d-band center (*ε*_d_), and the binding energy of subsurface hydrogen and carbon (*E*^sub^_b,H_ and *E*^sub^_b,C_, respectively).

The NP elemental, bulk, and surface properties were converted into materials-specific primary features by taking the composition average, indicated by the bar in 
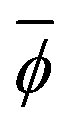
, where *ϕ* is an elemental, bulk, or surface parameter:1
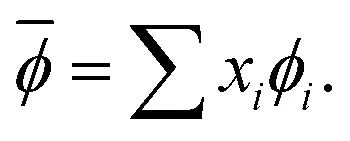
In eqn (1), *x*_*i*_ are the nominal molar fractions of each metal in the materials' composition. For instance, *x*_Pd_ = 1/10 and *x*_Ag_ = 9/10 for the material based on the supported Pd_1_Ag_9_ alloy. In total, 20 candidate descriptive parameters were collected, as shown in Table 1.

### 2.2 The SISSO approach

Starting from the primary features, the SISSO approach creates a large pool of analytic expressions, *e.g.*, containing millions of elements, by iteratively applying a predefined set of mathematical operators such as addition, multiplication, logarithm. Then, compressed sensing^68,69^ is used to identify the few analytical functions, that combined by weighting coefficients provide the best correlation between the selected primary features (genes) and the target property, here the ethylene selectivity *S*_C_2_H_4__. The SISSO model has the form2
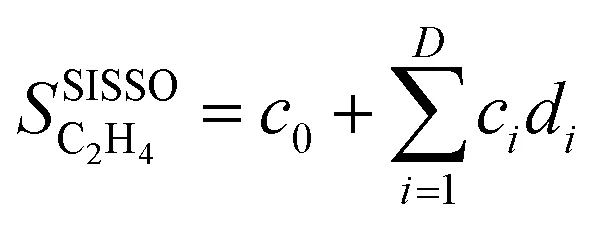
where *d*_*i*_ are the selected functions called *descriptors* and *c*_*i*_ are fitted coefficients. *D* is the number of selected functions and referred to as the dimensionality of the descriptor *vector*. The number of times the mathematical operators are applied (termed rung and denoted *R*) and *D* are hyperparameters of SISSO. They control descriptor complexity. In this work, we used the SISSO++^70^ code and considered the following mathematical operators, where *ϕ*_*i*_ and *ϕ*_*j*_ are two arbitrary features: *ϕ*_*i*_, *ϕ*_*i*_ + *ϕ*_*j*_, *ϕ*_*i*_ − *ϕ*_*j*_, *ϕ*_*i*_ × *ϕ*_*j*_, 
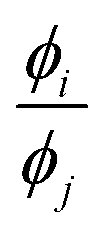
, *ϕ*_*i*_^−1^, exp(*ϕ*_*i*_), *ϕ*_*i*_^2^, *ϕ*_*i*_^3^, ln(*ϕ*_*i*_), *ϕ*_*i*_^6^, *ϕ*_*i*_^1/2^, *ϕ*_*i*_^1/3^, and exp(−*ϕ*_*i*_). The units of the primary features are respected so that, for instance, the operators addition and subtraction are only allowed between features with the same unit. We used a nonlinear optimization during the generation of expressions to include scale and bias terms for the mathematical operations logarithm and exponential.^39^

A nested five-fold cross validation was used in order to determine the hyperparameters of the SISSO model (*R* and *D*) and to estimate the predictive performance. In the outer loop of this cross-validation scheme, the dataset is randomly split into five folds with equal size. Each fold is then used as the test set once, while the remaining four folds are used to train a model. In the inner loop, a new five-fold split is done using the training sets obtained from the first split. Each fold obtained from this second split is used as a validation set once, while the remaining four folds are used as the training sets. The validation sets are used to determine the hyperparameters of the SISSO model. The hyperparameters are chosen such as the root-mean-squared errors (RMSEs) evaluated on the validation sets are minimized. For the modelling of *S*_C_2_H_4__, we identify *R* = 2 and *D* = 3 as appropriate hyperparameters. The test sets are used to estimate the predictive performance. The distribution of errors evaluated on the test sets indicate a good predictive performance for the modelling of *S*_C_2_H_4__. The mean test error value, for instance, is equal to 0.105. This value is below 25% of the standard deviation of the distribution of *S*_C_2_H_4__ values across the entire dataset. Further details on the application of the SISSO approach to model *S*_C_2_H_4__ (ref. 37) and on the nested cross validation^71^ are available elsewhere.

### 2.3 Sensitivity analysis

The models identified by SISSO generally only depend on a few of the initially offered primary features. However, the relative importance of these different genes that appear in a SISSO model might differ significantly. Sensitivity analyses identify the most influential input variables of a model.^70,72–75^ Thus, they facilitate the extraction of physical insight and help determining the most promising materials. Sensitivity analyses are referred to as local when they provide sensitivity scores per data point (*e.g.*, per material) or global when they provide average sensitivity scores for specific data space, *e.g.*, the entire population of materials. Additionally, the sensitivity analysis can be model specific or model agnostic. Model-agnostic sensitivity analyses examine how changes in an input variable affect the target (output) by systematically modifying the values of the input variables and recording the changes in the model output. The Sobol method, for instance, is a popular model-agnostic global sensitivity analysis^70,73,76^ that decomposes the variance of the model output into contributions from individual input variables and their interactions.

Here, we use the gradient-based partial-effects (PE) sensitivity analysis to assess the impact of genes identified by the SISSO approach. The PE method^48,49,77^ quantifies the impact of a given gene in the model's output when the remaining genes are fixed by means of the partial derivative of the model with respect to this gene.^49,78^ This model-specific approach exploits the analytical expressions identified by SISSO and provides local sensitivity scores. The local sensitivity analysis is insightful when the physical processes governing the target are significantly different for different groups of materials. In such cases, the contribution of a specific gene might be different for different materials. PEs are less computationally demanding than widely used analyses such as Sobol, since the partial derivative can be obtained analytically.

Let us now apply this concept to 
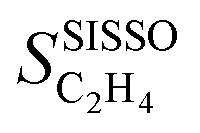
. The partial derivative with respect to gene *ϕ*_*j*_ is:3
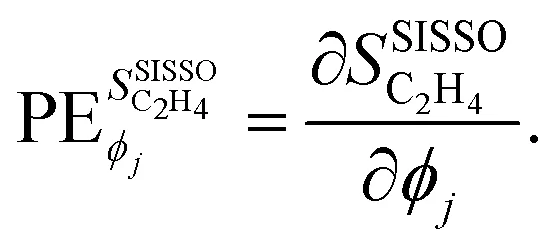
The higher the magnitude of the PE, the more sensitive is the model to the gene. The sign of the PE indicates whether the relationship between the gene and the target is (locally) directly or inversely proportional. 
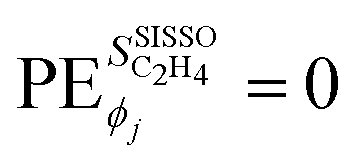
 if an initially offered primary feature *ϕ*_*j*_ was not selected in the SISSO model. The unit of 
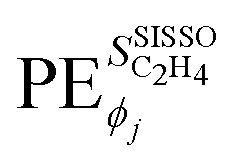
 is the unit of the target divided by the unit of the gene *ϕ*_*j*_. Thus, 
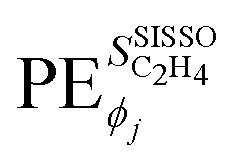
 values associated to genes having different units are not directly comparable. This is inconvenient when a quantitative comparison among PEs corresponding to different genes that appear in a given model is desired. In order to obtain PEs that can be directly compared across genes which might have different units and scales, we define scaled partial effects (SPEs) as4
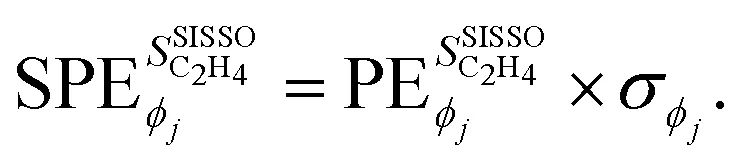
In eqn (4), *σ*_*ϕ*_*j*__ corresponds to the standard deviation of the distribution of *ϕ*_*j*_ values in the entire population, *e.g.*, of materials. In general, such population of materials is practically infinite and unknown. Thus, here we estimate *σ*_*ϕ*_*j*__ using the training data set, assuming that the distributions of primary features in the training set are appropriately described by *σ*_*ϕ*_*j*__. 
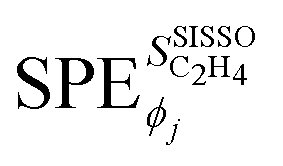
 has the unit of the target. Provided that the SISSO model expression 
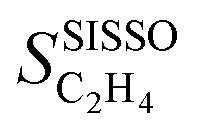
 is differentiable, the quantities defined in eqn (3) and (4) are analytical expressions obtained by analytical differentiation. The values of these expressions can be evaluated for each gene or, equivalently, for each data point, providing local sensitivity scores. Global sensitivity scores can then be derived by computing the SPEs for all data points and averaging the results:5
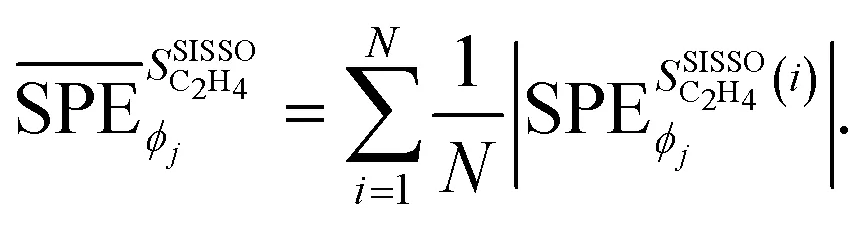
In eqn (5), 
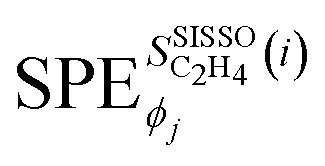
 are the values of the SPEs evaluated for the *i*-th entry of the training data set and the sum runs over the *N* entries of this data set. The derivatives of SISSO models were evaluated using the SymPy package.^79^ This paper introduces the PE-based sensitivity approach applied to SISSO models. More details about this PE approach and other applications in materials science will be discussed in an upcoming study.^80^

## 3 Results and discussion

This section summarizes the results for the nine mentioned NP catalysts. We analyze the evolution of ethylene selectivity *S*_C_2_H_4__ with time on stream *t*_OS_ during the hydrogenation of concentrated acetylene streams at 150 °C. Modeling the evolution of the catalytic performance with *t*_OS_ allows obtaining insights on the modifications that the materials experience at early stages of the reaction and it allows estimating the time span during which the material can be effectively utilized.

### 3.1 Measured ethylene selectivity

**Fig. 3:**
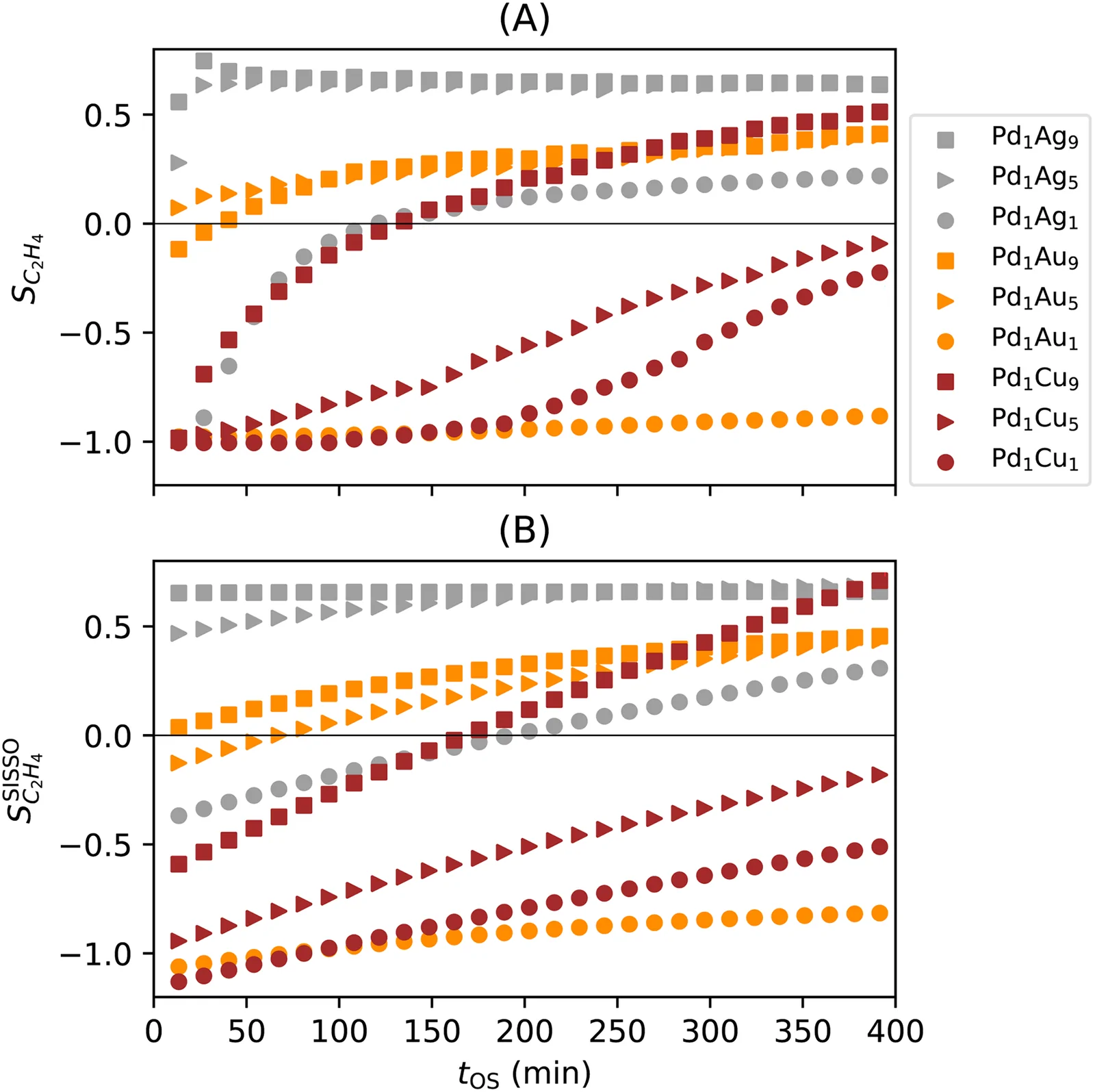
Experimental results and SISSO modelling for the ethylene selectivity in the selective hydrogenation of concentrated acetylene streams catalyzed by palladium-based alloys supported on alumina. (A) Measured *S*_C_2_H_4__ with time on stream. (B) Fit of the SISSO model of eqn (6)
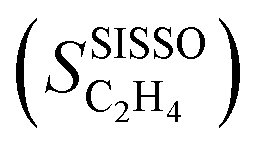
 to the data. Figure adapted from ref. 37.

The values of *S*_C_2_H_4__ range from −1 to 1, as ethylene is both a product of the acetylene selective hydrogenation reaction and part of the reaction feed. Negative *S*_C_2_H_4__ indicates that more ethylene from the feed is consumed than formed from acetylene. Positive *S*_C_2_H_4__ indicates selective materials and conditions. The desired behavior is *S*_C_2_H_4__ = 1 or, equivalently, 100% conversion. Diverse performance scenarios ranging from unselective to highly selective are observed among the considered materials and *t*_OS_ (Fig. 3A). The different catalysts show even qualitatively different performance. The highly selective situations present *S*_C_2_H_4__ values of up to 0.75. In general, the materials containing palladium–silver NPs are the most selective ones, followed by palladium–gold, and palladium–copper. Additionally, different profiles for the evolution of *S*_C_2_H_4__ with *t*_OS_ are observed depending on the material. Most of the materials based on the palladium–silver alloys are selective from the start of the reaction, *i.e.*, from *t*_OS_ = 0. For these materials, the selectivity reaches a stable level and then slightly decreases at longer *t*_OS_. In contrast, materials containing palladium–gold and, in particular, palladium–copper NPs present constantly increasing *S*_C_2_H_4__ with *t*_OS_. The initial selectivity of Pd_1_Cu_9_, *e.g.*, is *S*_C_2_H_4__ = −0.89 but it increases with *t*_OS_ up to *S*_C_2_H_4__ = 0.47 after 365 min. This represents a drastic shift from a completely unselective condition to a highly selective one.

### 3.2 SISSO model for ethylene selectivity

SISSO was applied to the training dataset containing 539 data points, associated to the 9 materials measured at multiple *t*_OS_'s. This dataset contains the measured target *S*_C_2_H_4__ and 20 primary features, including *t*_OS_ as well as a wide range of measured and calculated parameters characterizing the entire materials set and the chemistry of the bulk and surface of the NPs (Table 1). The choice of some of the offered primary features reflects relevant physical processes identified by previous surface science studies and atomistic simulations on model systems. The selectivity towards ethylene depends on the competition between ethylene desorption and its hydrogenation to ethane. Thus, the d-band center^17^ is included as a primary feature, as it correlates with the adsorption of ethylene as well as other intermediates of the acetylene hydrogenation reaction such as vinylidene and ethyl.^81,82^ Additionally, we offer primary features that capture the interaction of NP surfaces with hydrogen, since the availability of surface and subsurface hydrogen impacts the rate of ethylene hydrogenation to ethane.^83^ Primary features reflecting the interaction of metal surfaces with carbon are also included, as the formation of surface carbides has been shown to be a crucial factor determining the selectivity in palladium systems,^57,84–90^ as it limits the hydrogen availability. Further details on the dataset and SISSO approach are given in the Methods section.

The good quality of the fit provided by the SISSO model for ethylene selectivity identified in ref. 37, denoted 
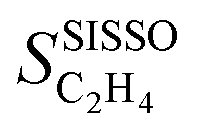
, is shown in Fig. 3B. This model is able to describe the selectivity of the different materials and its evolution with *t*_OS_. The expression of the model is6
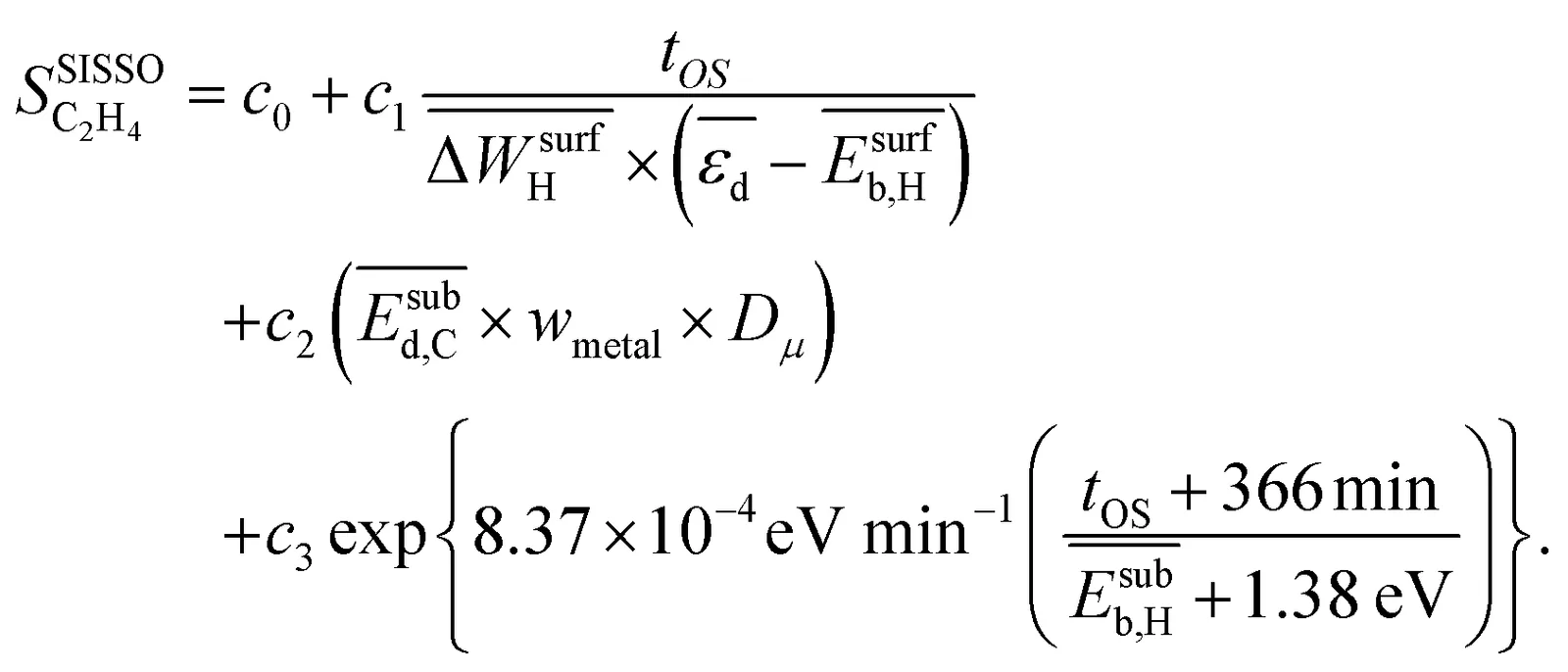
This model is based on a 3-dimensional descriptor vector. In eqn (6), *c*_0_ = −0.098, *c*_1_ = −1.22 × 10^−6^ eV^2^ min^−1^, *c*_2_ = 48.491 eV^−1^ nm^−1^, *c*_3_ = −2.65. The model expression contains *t*_OS_, required to describe the time dependency. Additionally, the total metal loading (*w*_metal_) and mean value of particle size (*D*_*µ*_), measured prior to the catalytic test, are also selected by SISSO as key experimental genes in eqn (6). Finally, the following calculated genes are part of eqn (6): the average d-band center 
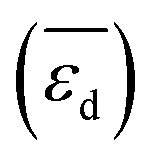
, the average deformation energy of subsurface carbon 
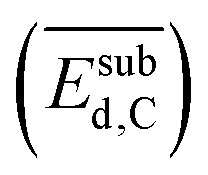
, the average work-function shift with hydrogen adsorption 
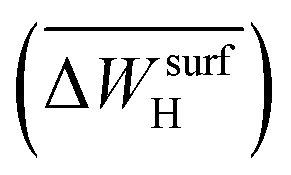
, the average binding energy of hydrogen 
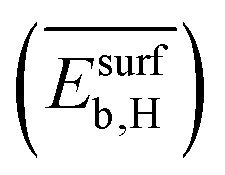
, and the average binding energy of subsurface hydrogen 
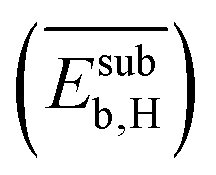
. From the initially offered 20 primary features, SISSO selected 8 relevant genes to describe *S*_C_2_H_4__ (eqn (6)). However, the genes that enter in the model expression can impact the model to different extents. In order to identify the most influential genes of eqn (6), the PE analysis is applied.

### 3.3 Sensitivity analysis of the SISSO model for ethylene selectivity

**Fig. 4:**
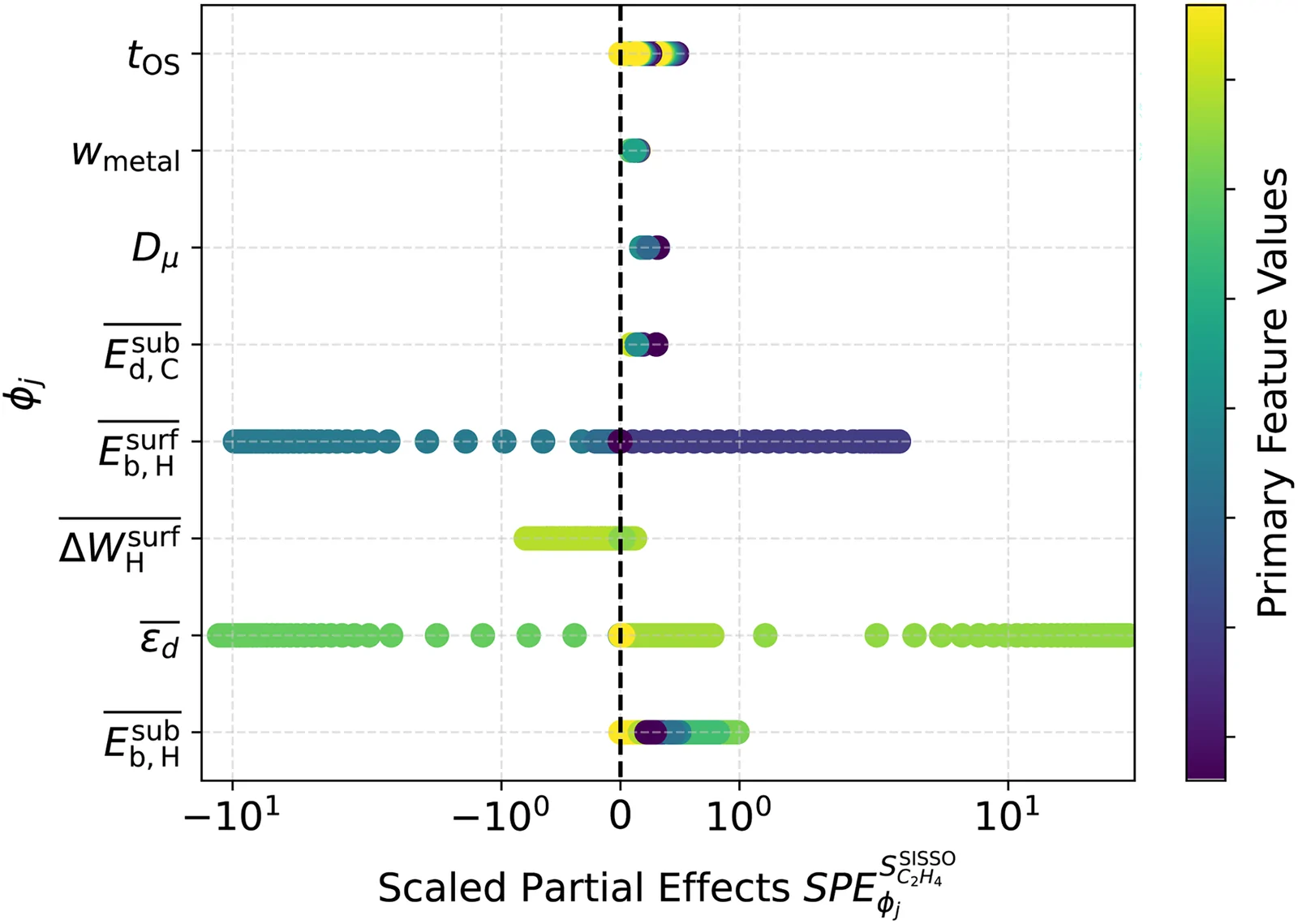
Sensitivity analysis for the SISSO model of ethylene selectivity 
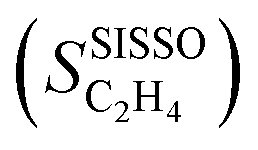
 in the selective hydrogenation of concentrated acetylene streams catalyzed by palladium-based alloys supported on alumina. The scaled partial effects 
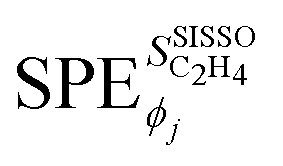
 quantify how influential the genes *ϕ*_*j*_ of the model 
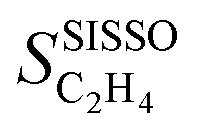
 are. Each circle corresponds to one data point, associated to one material and one time-on-stream value. The color scale indicates the value of each gene.

The 
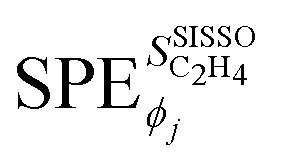
’s for the 8 genes that were selected by SISSO in the expression of the model 
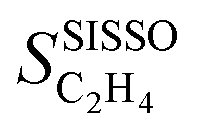
 are shown in Fig. 4. In this figure, each line corresponds to one gene and each circle corresponds to the 
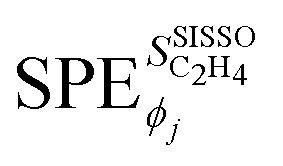
 evaluated for one data point of the training dataset. The circles are colored according to the values of genes. The scale of the *x* axis is logarithm for a better visualization, as the SPEs have different magnitudes. For primary features that were not selected by SISSO in eqn (6), 
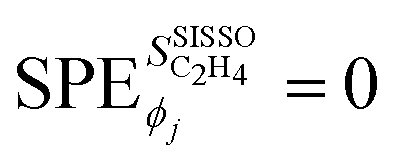
. Details of the PE analysis are available in the Methods section.

In order to assess the overall sensitivity of the 
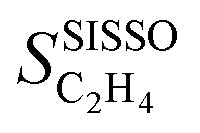
 model with respect to the genes, we evaluated the mean absolute values of SPEs 
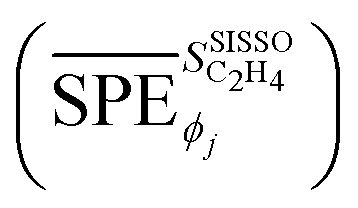
 for each gene as a global sensitivity score. The 
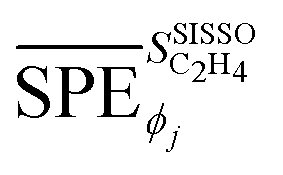
 values are 0.15, 0.12, 0.23, 0.16, 0.75, 0.092, 2.81, and 0.42 for *t*_OS_, *w*_metal_, *D*_*µ*_, 
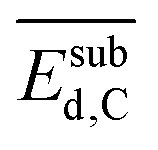
, 
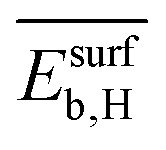
, 
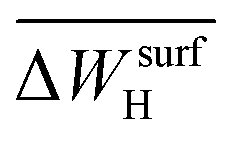
, 
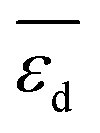
, and 
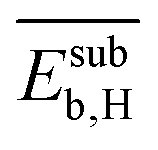
, respectively. Therefore, the relative global influence of the genes to the model decreases as 
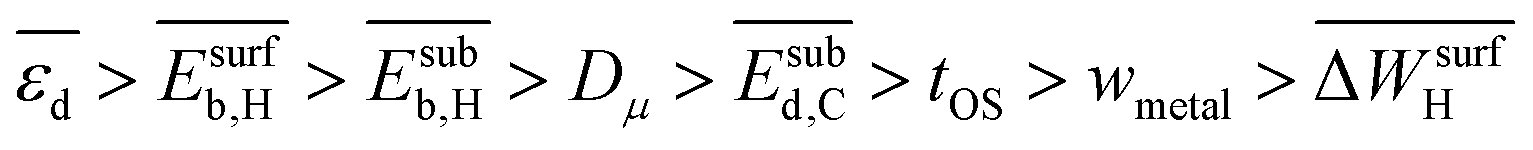
. The most influential gene in the 
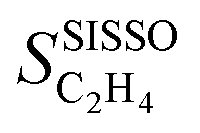
 model (eqn (6)) is the average d-band center 
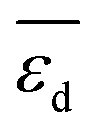
. This can be related to the key role of physical processes related to the adsorption of reaction intermediates on the NP surface. As shown previously, the alloying of palladium with a second metal downshifts the d-band center, weakening the adsorption of π-bonded species such as ethylene, for instance.^81,82^ Thus, ethylene desorption is favored over its hydrogenation to ethane. The second and third most influential genes are 
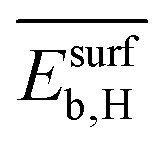
 and 
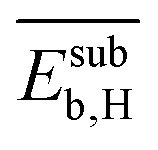
. They reflect the bond strength between the surface and subsurface with hydrogen. Thus, their high relevance can be related to the availability of surface and subsurface hydrogen and its impact on the rate of hydrogenation of ethylene to ethane, which hinders the formation of the selective-hydrogenation product ethylene.^91^ We note that the values of 
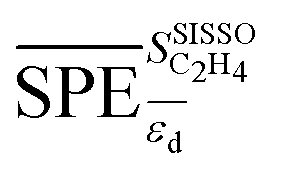
 and 
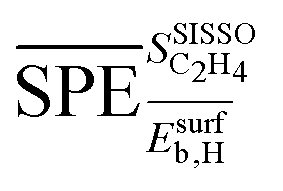
 can be positive or negative depending on the material and *t*_OS_ (Fig. 4), indicating that the relationships between 
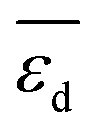
 or 
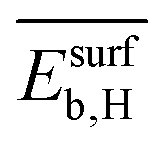
 and *S*_C_2_H_4__ can be directly or inversely proportional. Thus, the adsorption properties can either favor or hinder the selectivity. Finally, the fourth most influential gene *D*_*µ*_ could be related to a particle size effect on the ethylene selectivity. Indeed, the size of palladium or copper NPs can significantly affect their selectivity in the hydrogenation of unsaturated bonds by controlling the relative amount of corner and edge sites, which are less coordinated and can thus provide stronger adsorption than the surface sites on closely packed surfaces.^87,92,93^ The most influential genes identified by the sensitivity analysis confirm the crucial role of the adsorption of π-bonded species and hydrogen in modulating the ethylene selectivity, as discussed in previous works addressing the hydrogenation of diluted acetylene streams.^81,82^ Additionally, the PEs identify a structure-sensitivity effect, which was only discussed in detail for the hydrogenation of dienes.^87,92,93^ The 
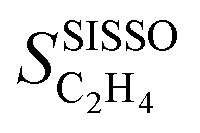
 model is less sensitive to the genes 
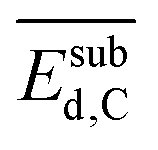
, *t*_OS_, *w*_metal_, and 
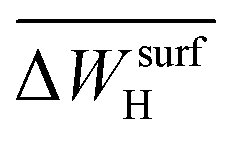
. Thus, the PE analysis indicates a low influence of (sub)surface carbon on the ethylene selectivity for the systems studied here. This indicates that the formation of surface carbides discussed previously in the context of the hydrogenation of diluted acetylene streams might be less relevant to determine the selectivity for the systems considered here.^57,84–90^

**Fig. 5:**
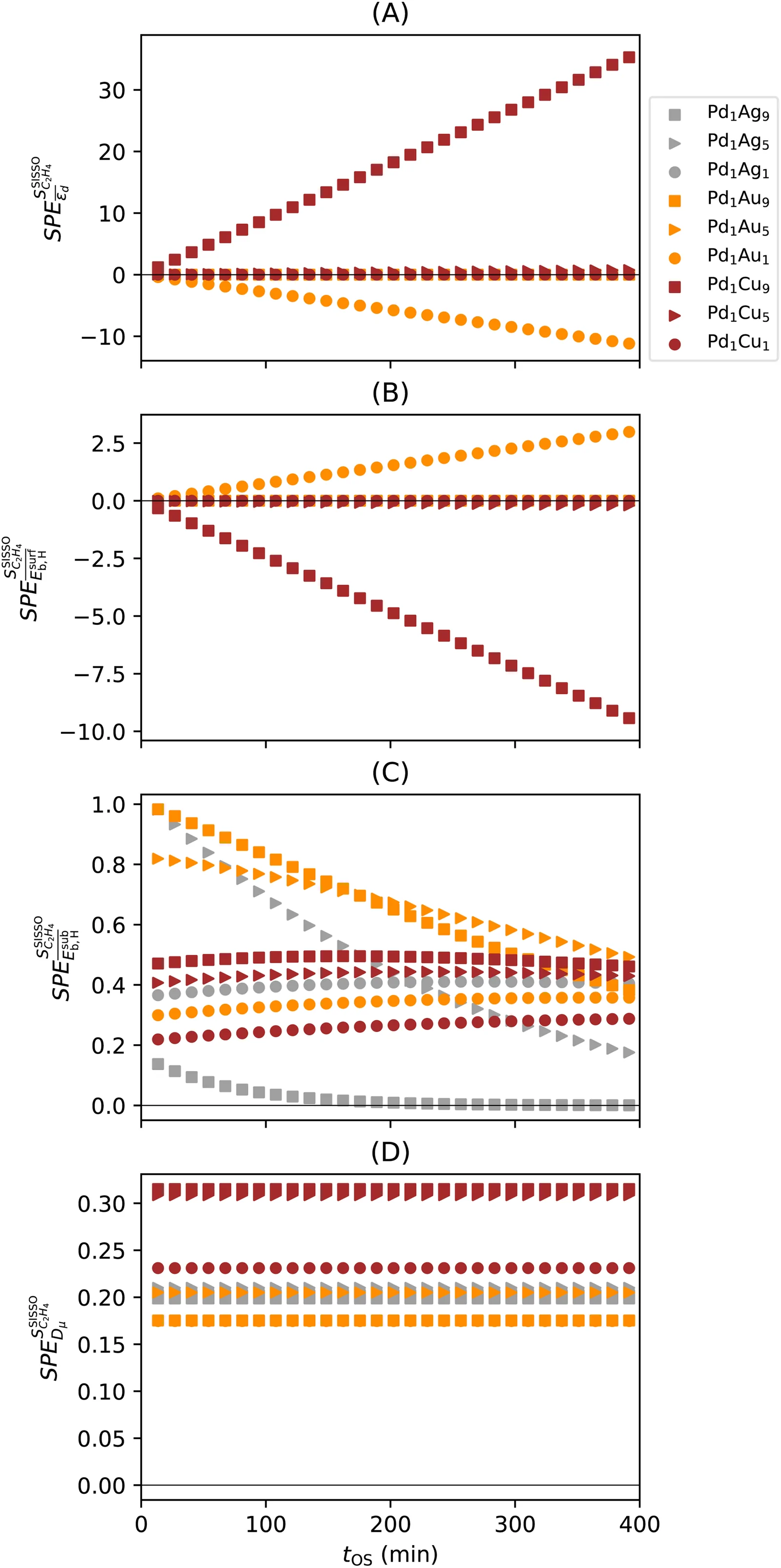
Evolution of the scaled partial effects 
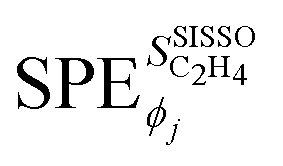
 with time on stream (*t*_OS_) for the four most influential genes selected by SISSO in the model of eqn (6)
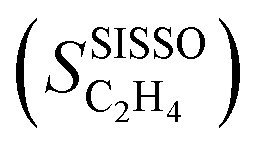
: 
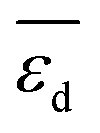
 (A), 
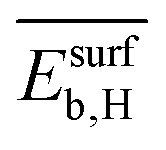
 (B), 
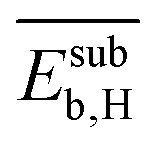
 (C), *D*_*µ*_ (D).

Fig. 5 shows the SPEs for the 4 most influential genes for each of the materials as a function of *t*_OS_. The magnitude of the SPE scores associated to the genes 
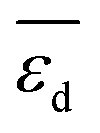
 and 
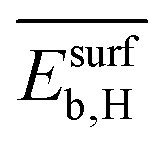
 is higher for the material based on Pd_1_Cu_9_ compared to the remaining materials. This difference increases with *t*_OS_. This suggests that the impact of physical processes related to adsorption on *S*_C_2_H_4__ is particularly important for this material, especially at long *t*_OS_. It can be related to the fact that the Pd_1_Cu_9_ alloy presents the most drastic change in *S*_C_2_H_4__ with time, as it is a total hydrogenation catalyst at *t*_OS_ = 0 min, but it becomes selective at longer reaction times (Fig. 3). Interestingly, whereas 
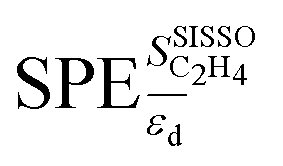
 is positive and thus an increase in 
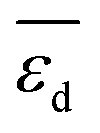
 favors selectivity for Pd_1_Cu_9_, 
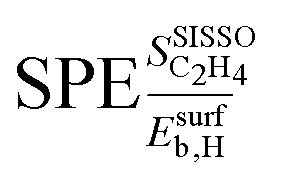
 is negative and an increase in 
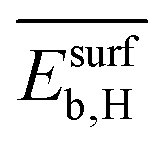
 hinders the selectivity. The SPEs related to the gene 
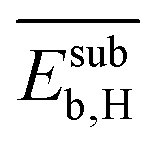
 are relatively insensitive to *t*_OS_ for most materials. However, the 
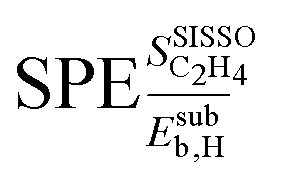
 values decrease significantly with *t*_OS_ for the materials based on Pd_1_Au_9_, Pd_1_Au_5_, and Pd_1_Ag_5_, indicating that physical processes related to subsurface hydrogen are only relevant at the beginning of the induction period for these materials. Finally, the analysis of per-material 
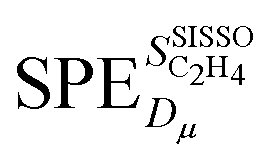
 scores shows that the particle-size distribution impacts the copper-based materials, in particular the materials based on Pd_1_Cu_9_ and Pd_1_Cu_5_, to a greater extent compared to the remaining materials. This indicates a higher influence of structure-sensitivity for copper-based catalysts. This analysis illustrates how per-data-point, *e.g.*, per-material, SPE scores can be used to obtain insights on the most relevant underlying physical processes for specific data points. In addition to providing global and local, per-data-point physical insights *via* the identification of the most influential genes in the SISSO model, the sensitivity analysis can also be used to design high-throughput materials-screening protocols.^70^ In these protocols, the values of the most influential genes are used to determine rules indicating new materials likely presenting desired target property values, *e.g.*, high values.

The three most influential genes identified by the sensitivity analysis 
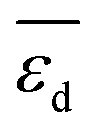
, 
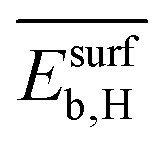
, and 
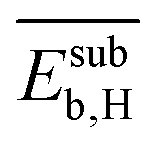
 are parameters calculated *via* DFT-GGA calculations for model systems and they highlight the importance of the adsorption properties for catalysis. However, the relevance of the experimental parameter *D*_*µ*_ stresses the utility of measured primary features to capture aspects that can hardly be included in the theoretical description. Indeed, the distribution of NP sizes is influenced by the properties of the metal alloy as well as by metal–support interactions and synthesis conditions. Additionally, this distribution might be also modified under the reaction conditions. Modelling all such physical processes by theory is unfeasible. The utilization of materials properties measured under conditions close to reaction conditions, *e.g.*, using *in situ* and *operando* spectroscopy, as primary features was also shown to be crucial in order to capture the catalyst restructuring effect on the catalytic performance.^2,34^ Therefore, the combination of experimental and theoretical (calculated) primary features is a promising avenue for the identification of descriptors that capture the complexity of heterogeneous catalysis.^36^ As systematic experimental and theoretical data become available,^20,94–96^ the materials-genes concept offers a framework for integrating surface science, atomistic modelling, and *operando* characterization towards efficient catalyst design.

Finally, we stress that SISSO and the materials genes will only provide reliable descriptions for materials and reaction conditions governed by the same underlying physical processes that govern the performance of the situations in the training set. The reliability of the SISSO models can be assessed and used in order to systematically acquire new data to improve the description for portions of the data space that are not sufficiently covered by the training dataset.^97^ This aspect is discussed in detail in the contribution by Nair *et al.*

## 4 Conclusions

In this contribution, we propose rethinking the description of heterogeneous catalysis by focusing on statistical correlations identified in experimental and theoretical data generated systematically rather than explicitly modeling all the underlying physicochemical processes and their intricate interplay *via* atomistic simulations. This approach can capture the intricacy of catalysis more effectively than previous computational approaches, as it does not assume a single underlying physical model. By applying the SISSO approach with the gradient-based PE sensitivity analysis, we identified the most influential basic physicochemical parameters correlated with the selectivity of metal NPs supported on alumina and applied in the hydrogenation of concentrated acetylene streams. These parameters indicate that the adsorption of hydrogen and of molecules containing π bonds modulate the ethylene selectivity. Additionally, a structure-sensitivity effect on the selectivity is captured by the experimentally measured NP size. In addition to the chemical insights, the materials-genes concept enables the design of improved materials and reaction conditions for catalysis.^37^ In particular, the most influential physicochemical parameters determined by sensitivity analyses can be used to efficiently screen new candidate materials.^70^ The integration of materials genes with automated experiments and large-scale atomistic simulations offers a promising path towards more predictive and generalizable catalyst design.

## Conflicts of interest

The authors declare no competing interests.

## Supplementary Material

FD-267-D5FD00137D-s001

## Data Availability

The dataset is available in the Excel supplementary information file of ref. 37. The SISSO analysis is available at https://github.com/lfoppa/Focused-AI-SGD-SISSO-acetylene-hydrogenation.
