# Supplementary figures and images for: Rethinking catalysis: interpretable AI and description of real-world conditions via materials genes
Source: Faraday Discuss. 2026 Feb 6. Online ahead of print. doi: 10.1039/d5fd00137d (PMC13151911; doi:10.1039/d5fd00137d)

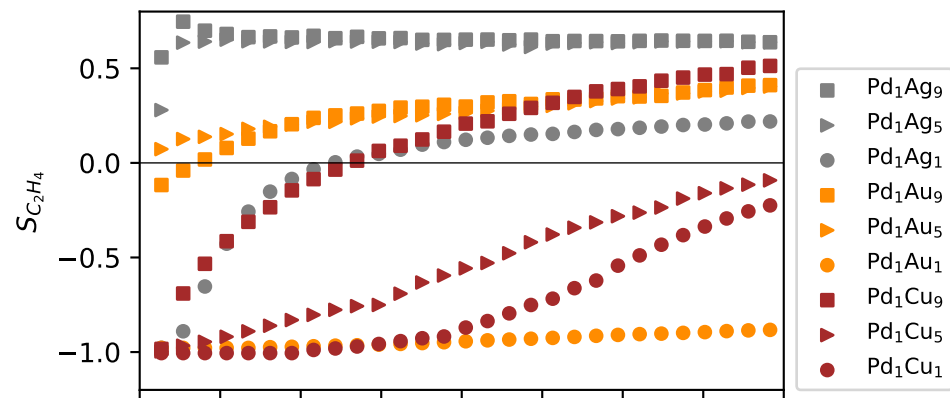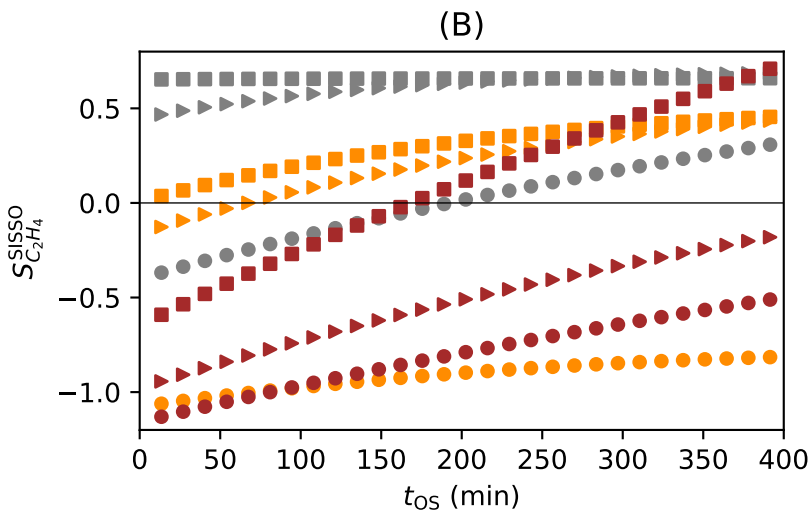

Supplement: FD-OLF-D5FD00137D-s001 [file FD-OLF-D5FD00137D-s001.pdf]
